# Mechanotransduction Mechanisms in Mitral Valve Physiology and Disease Pathogenesis

**DOI:** 10.3389/fcvm.2017.00083

**Published:** 2017-12-22

**Authors:** Leah A. Pagnozzi, Jonathan T. Butcher

**Affiliations:** ^1^Meinig School of Biomedical Engineering, Cornell University, Ithaca, NY, United States

**Keywords:** mitral valve, valve disease, mechanotransduction, pathogenesis, biomechanics

## Abstract

The mitral valve exists in a mechanically demanding environment, with the stress of each cardiac cycle deforming and shearing the native fibroblasts and endothelial cells. Cells and their extracellular matrix exhibit a dynamic reciprocity in the growth and formation of tissue through mechanotransduction and continuously adapt to physical cues in their environment through gene, protein, and cytokine expression. Valve disease is the most common congenital heart defect with watchful waiting and valve replacement surgery the only treatment option. Mitral valve disease (MVD) has been linked to a variety of mechano-active genes ranging from extracellular components, mechanotransductive elements, and cytoplasmic and nuclear transcription factors. Specialized cell receptors, such as adherens junctions, cadherins, integrins, primary cilia, ion channels, caveolae, and the glycocalyx, convert mechanical cues into biochemical responses *via* a complex of mechanoresponsive elements, shared signaling modalities, and integrated frameworks. Understanding mechanosensing and transduction in mitral valve-specific cells may allow us to discover unique signal transduction pathways between cells and their environment, leading to cell or tissue specific mechanically targeted therapeutics for MVD.

## Introduction

The mitral valve is a bicuspid valve that facilitates the flow of blood from the left atrium to the left ventricle. Mitral valve disease (MVD) affects 2.4% of the population and is a common congenital heart defect (1, 2). In adults, the most common disorders of the valve are mitral insufficiency (i.e., regurgitation), mitral stenosis, myxomatous degeneration, and mitral valve prolapse, with broad disease likely to include several of these effects.

The mitral valve leaflets consist of four layers that differ in extracellular matrix (ECM) composition and mechanical properties. The thickest layer of the valve, the fibrosa, is the main load bearing layer. It provides the majority of leaflet tensile strength through a thick layer of dense, aligned collagen fibers while a looser collagen network with increased glycosaminoglycan (GAG) and proteoglycan content provides compressive strength. The mitral valve is mechanically supported through the GAG-rich chordae tendinae which attach the mitral leaflets to the papillary muscles along the ventricular wall and maintain valve closure during systole. MVD results in altered mechanical and structural properties of the valve. Myxomatous mitral valves are characterized by leaflet enlargement, annular dilation, thickened and elongated chordae, GAG accumulation, loss of structure, increased compliance, and myxoid lesions. Disorganization and remodeling of the ECM and weakening of the chordae result in a loss of most of the valve’s mechanical properties and an overall thickened and enlarged leaflet. This in turn prevents the valve from fully closing causing symptoms of mitral regurgitation and prolapse.

The mitral valve is a dynamic structure which changes mechanically during the cardiac cycle; the constant flow of blood and opening and shutting of the valves exposes the tissue to a complex and demanding environment. The valve is subjected to bending, deformation, large area changes, shear stress, and heterogeneous strains in response to myocardial contraction, transvalvular pressure, and hemodynamic flow. The mitral valve exhibits a non-linear stress–strain relationship with complex viscoelastic and axial coupling behaviors (3, 4). These dynamic and adaptive interactions between the myocardial wall and valve leaflets ultimately impact the mechanical stress and strain experienced by cells through the ECM (5).

Cells and their ECM exhibit dynamic reciprocity, continuous, bidirectional interaction between cells and their ECM, in the growth and formation of tissue through mechanotransduction, the conversion of mechanical signals into biochemical responses (6). Cells and their ECM reorganize *via* a complex of mechanoresponsive elements (6) to physically regulate the spatiotemporal distribution of biochemical components maintaining homeostasis. MVD has been linked to a variety of mechano-active genes, such as extracellular components, mechanotransductive elements, and transcription factors. Mechanical stimulus in the microenvironment provides inductive signals of homeostasis and remodeling to the native cells—valve interstitial cells (VICs), and endothelial cells (VECs).

Valve endothelial cells reside on the exterior of the valve, maintain a non thrombogenic surface layer, and regulate immune and inflammatory reactions. The majority of valve cells are the VICs, a mesenchymal population that resides in all layers of the valve, distinct in their ability to differentiate into multiple phenotypes. There are five known phenotypes of VICs: embryonic progenitor endothelial/mesenchymal, quiescent, activated, progenitor, and osteoblastic VICs which may convert from one form to another. Most VICs in the healthy adult valve are quiescent with a small population of activated VICs to maintain base ECM remodeling. In pathological states, there is an increase in activated VICs which regulate repair and remodeling, which may lead to fibrosis and calcification. Inflammation, biochemical, and mechanical stimuli can induce activation of quiescent fibroblasts into myofibroblasts. VICs and VECs continuously remodel their environment by secreting and degrading ECM, and adapting their gene, protein, and cytokine expression to alter phenotype and function. These dynamic and adaptive interactions between the myocardial wall, flowing blood, and valve leaflets ultimately impact the mechanical stress and strain experienced by cells through the ECM. The movement, anisotropic deformation, and complex geometries of the mitral valve create a variety of ever changing mechanical cues between the cells and their matrix. ECM composition, fiber alignment, and compaction regulate cell deformation and thus mechanotransductive response. By focusing on broad classes of mechanosensing pathways as well as their integration in mechanotransduction, this review will explore the biomechanical mechanisms at play in the mitral valve microenvironment and mediators of mechanotransduction in this tissue.

## Mechanobiology of Mitral Valvulogenesis

During valve development, the embryonic heart transforms from a myocardial tube into a complex, four chambered, mature structure. Valve cells differentiate from endocardial cells during gastrulation and by E9.5 valvulogenesis begins when the heart tube loops creating the primitive ventricle and atria. In these early embryos position sensing (7, 8) and force transduction instruct lineage allocation. Endothelial cells (ECs) of the endocardium form valve cushions in a GAG-rich cardiac jelly where, in response to growth factors, such as Transforming Growth Factor-β (TGF-β), they undergo endothelial to mesenchymal transition (EMT). ECs reorganize their actin architecture to permit migration, adhesion, and morphogenesis in the embryo. Knockout of cytoskeletal adaptors in ECs causes disorganized cytoskeletal organization, cell morphology, impaired focal adhesion development, and actin signaling, inhibiting EMT in embryonic mice (9). Atrioventricular endocardial cells adopt a cuboidal morphology prior to EMT which seems mediated by cardiac contraction- in mutants which lack heart contraction, endocardial cells fail to change shape and initiate EMT (10).

During EMT cell–cell contacts are downregulated and processes governing cell–matrix adhesions and cytoskeleton reorganization are upregulated (11). Cells acquire an invasive phenotype, allowing them to migrate into the cardiac jelly, degrade hyaluronan, and deposit collagen, versican, and proteoglycans to form mature leaflets. Cushion mesenchymal cells give rise to VICs post-EMT which organize their surrounding matrix into a fibrous, rigid tissue able to withstand the hemodynamic loading of the beating heart. Contractile VICs condense the ECM by pulling on it, creating cell–matrix alignment in response to mechanical cues (12, 13). During valvulogenesis, tension points are created which may promote the secretion and alignment of collagen fibrils from VICs in a manner similar to that seen during tendon development (14).

Mechanotrasnduction of hemodynamic shear and strain are crucial to valvulogenesis. In zebrafish embryos knockdown of oscillatory flow sensitive gene klf2a results in dysfunctional or absent leaflet formation despite no change in retrograde flow (15). klf2a is related to signaling through mechano-sensitive ion channels, which is discussed later in the Ion Channel section. Physical occlusion of the inflow or outflow tract in zebrafish embryos results in hearts with an abnormal third chamber, looping defects, and impaired valve formation. In the embryo, red blood cells themselves generate important shear fluctuations different than that of normal hemodynamic shear which may mechanically influence ECs (16). Zebrafish with transvalvular flow alterations fail to undergo atrioventricular valve maturation from two to four leaflets despite no alterations in contractility (17). Tissue strain from variations in pressure and cardiac contraction also mechanically drive valve formation in a similar fashion to cell–cell and cell–matrix contacts. Mutations that inhibit myocardial contractility in the embryo fail to form cushions with chemical inhibition of contraction inhibiting endocardial ring formation in a dose dependent fashion (18). Cytoskeletal adaptors in embryonic ECs mediate actin dynamics, and mutations in them disrupt EMT and valvulogenesis (19). The impact of strain alterations are time dependent as altered cardiac preload results in morphological defects in zebrafish embryos treated in earlier and later developmental stages without impacting groups treated at 30–36 h post fertilization (20). Alterations in cell–matrix homeostasis later in life may reactivate physical or chemical cues of valvulogenesis, particularly EMT, causing aberrant elongation, remodeling, and stiffening (21, 22).

## Adherens Junctions and Cadherins

Adherens junctions are located at cell–cell contact points where they mediate cell adhesion, force, and signal transduction. Cells send out a finger-like lamellipodia to neighboring cells, which are stabilized by the acceptor lamellae with actin-myosin contractility. This actin finger determines the location and shape of the adherens junction and is co-localized with stress fibers in the neighboring cells. Adhesions are formed through integrin and cadherin interactions in both VECs and VICs at cell–cell and cell–integrin junctions, respectively. At adhesions, adhesion receptors interact with F-actin and adhesion proteins to regulate signaling, junction assembly, and maintenance. While traditionally recognized as distinct structures, adherens junctions and focal adhesions are intracellularly linked to the actin cytoskeleton, and activate the same signaling proteins and actin regulators (23).

Vinculin is a cytoplasmic actin binding protein, enriched at both cell–cell and cell–matrix adhesions, which regulates integrin dynamics and adhesion, stimulating polymerization, and remodeling through actin binding. Vinculin arranges itself in three domains: an integrin signaling layer, actin binding and force transducing layer, and actin regulatory layer. Vinculin is in an open active form in focal adhesions and a closed, inhibited form within the cytoplasm. In this inhibited form, the vinculin head domain interacts extensively with its tail in the integrin signaling layer and when these head–tail interactions are relieved (24), it migrates to the actin binding layer where it recruits proteins to regulate focal adhesion dynamics and cell migration (25).

Cadherins are calcium-dependent cell adhesion proteins composed of an extracellular region, a transmembrane domain, and cytoplasmic region. Cadherins connect the cortical actin cytoskeleton of neighboring cells and create zipper-like structures to maintain stable intercellular adhesion by regulating cortical tension and maintaining mechanical coupling between cells (26). In confluent monolayers, VICs with strong cell–cell contacts show weak expression of myofibroblastic marker α-smooth muscle actin (αSMA), suggesting cell contact inhibits myofibroblastic activation (27). In these conditions, cadherin protein complexes β-catenin and N-cadherin expression are decreased or absent (27). In aortic valve disease and development cell junction protein cadherin-11 (Cad-11) has been implicated in a variety of mechano-active defects and similar mechanisms may be at play in MVD. Cad-11, a known mediator of dystrophic calcification in calcific aortic valve disease, is strongly expressed in human calcified aortic leaflets with nodule formation dependent on strong cell–cell contacts (28) while cyclic strain upregulates Cad-11 and αSMA expression (29) in aortic VICs (AVICs). In canines with myxomatous valve disease, VE-cadherin was significantly decreased (30). Downregulation of VE-cadherin results in endothelial migration and EMT in zebrafish valvulogenesis (31) so similar expression in canines suggests a pathological proliferative and migratory endothelial phenotype (30).

Plakophilin-2 links cadherins to intermediate filaments in the cytoskeleton. In prolapsed mitral valves, increased Cad-11, N-cadherin, and aberrant presence of plakophilin-2 at the adherens junction, promotes latent TGF-β activation and pathological ECM remodeling (32). Cad-11 is expressed in chick mitral valves during development at the leaflet tips in endocardial cushion mesenchymal cells (31) and throughout the leaflets of remodeling valves in adults. In hyperlipidemic mice, Cad-11 expression was significantly increased in the aortic and mitral valves (33) inducing ECM remodeling and calcific nodule formation (34).

## Integrins

Integrins regulate and respond to force by connecting the ECM to the cytoskeleton. Composed of an α and β subunit which combine to approximately 24 unique heterodimers (35, 36), integrins bind to different ECM proteins and interact with cell-surface ligands, transmembrane proteins, proteases, and growth factors (37). Integrins receive and transmit signals from both sides of the plasma membrane (38, 39). Cytoskeletal contractions pull on integrin links to the matrix, deforming binding proteins that connect actin to focal adhesion proteins and integrin to arginine–glycine–aspartate (RGD) containing proteins, altering gene and protein expression (40). RGD is the main integrin binding domain in ECM proteins common to the mitral valve: collagens, laminin, fibrillin, and fibronectin (41, 42).

Several adhesive peptides control integrin-mediated cell adhesion. VICs strongly express the α2 and β1 subunits and α5β1 integrin (43, 44) Collagen I mimetic DGEA binds integrin α2β1 and promotes adhesion and ECM deposition in VICs (45). The α2β1 integrin is necessary in coupling VICs to collagen I, propagating VIC contraction into leaflet force generation (46). In combination with RGD, peptide VAPG with affinity to laminin and elastin, along with DGEA downregulate myofibroblastic and osteogenic differentiation in VICs (45). Blocking integrin receptor 67LR, with affinities to laminin and elastin, resulted in formation of calcific nodules (47) suggesting an anticalcific effect in binding. Disruption of VIC binding *via* the α_5_β_1_ integrin or the 67-kDa laminin receptor had a dramatic calcification-stimulating effect. Binding *via* the α_2_β_1_ integrin did not alter calcification or VIC phenotype; blocking α5β1 resulted in calcification in AVICs (43) and is likely to have similar pathology in mitral valves.

Integrins bind to and activate TGF-β, which modulates cell growth, adhesion, migration, and ECM synthesis (48, 49). TGF-β secretion consists of three proteins: TGF-β, latency-associated protein (LAP), and latent TGF-β binding protein (LTBP), an ECM-binding protein. Several integrins activate latent TGF-β through binding to an RGD integrin binding site on LAP (50). Under high stress, TGF-β controls expression of αSMA, stress fiber formation, and differentiates quiescent fibroblasts into contractile myofibroblasts creating a positive feedback cycle (51). Mechanically conditioning ECM releases active TGFβ1 (52) demonstrating the role of force in fibroblast activation. VICs grown on stiff surfaces have strong cell-ECM adhesions, contractility, and myofibroblast differentiation (53). Shear flow induces TGFβ1 production and myofibroblast differentiation of fibroblasts in collagen gels (54). In both embryonic and adult VICs, a quiescent phenotype is maintained in unstressed collagen hydrogels; however, contractile expression, TGF-β, and matrix remodeling are upregulated in response to tension (55).

**Figure 1:**
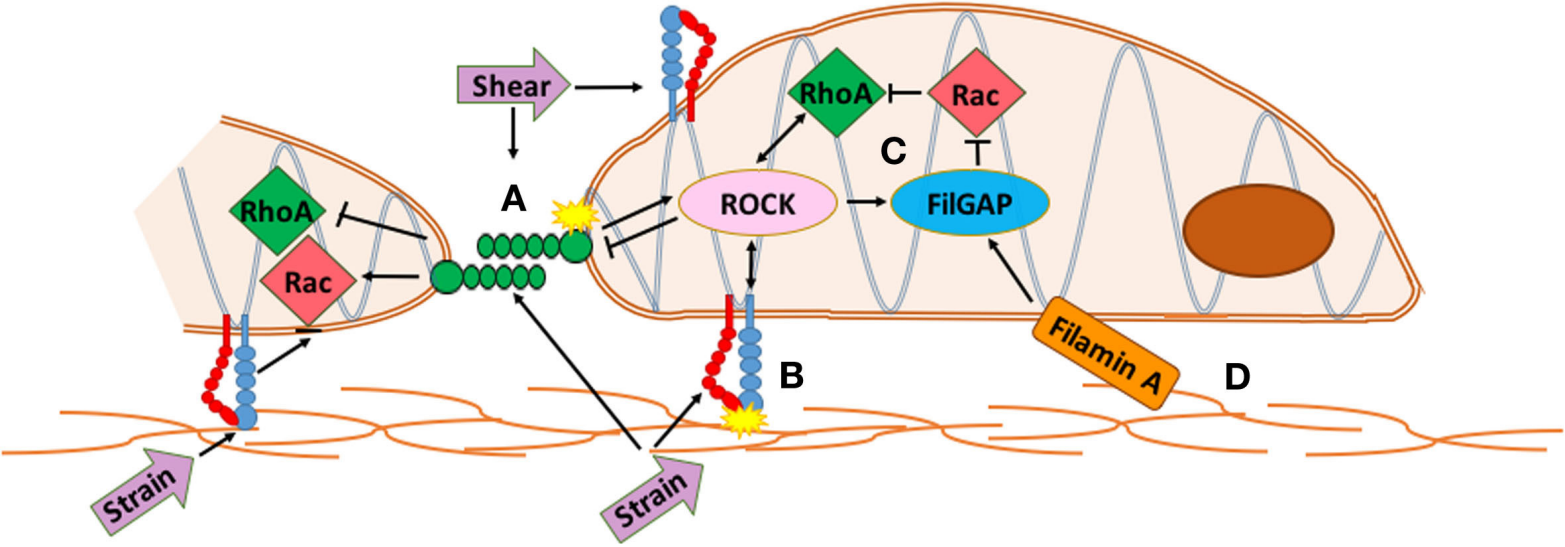
Integrated mechanotransduction of cadherins and integrins through the cytoskeleton and small GTPases. A. At cell–cell adhesion points coupled cadherins transduce strain and shear force into cytoskeletal remodeling and downstream signaling pathways. B. At cell–extracellular matrix (ECM) adhesions, strain is transduced through integrins into cytoskeletal remodeling and downstream signaling pathways. C. RhoA and Rac are mechano-sensitive small GTPases common to multiple methods of mechanotransduction in the mitral valve that act opposite and complementary to control cell migration, differentiation, and proliferation. RhoA regulates actin cytoskeleton and stress fiber formation while Rac1 regulates cell–cell adhesion, actin polymerization, lamellae protrustion, and cytoskeletal polarity. ROCK interacts with integrins and cadherins to mediate RhoA and Rac activity while FilGAP binds to filamin-A to control actin at cytoskeletal interfaces. D. The extracellular matrix interacts with the actin network directly through specific ECM components or through integrins and cadherins. See Section “Integrated Mechanotransduction” for more information.

Latent TGF-β binding proteins interact with fibrillin, a large structural protein that polymerizes into extracellular microfibrils and contributes to the functional integrity of connective tissue (56). Mutations in fibrillin-1 cause Marfan Syndrome (MFS) and related disorders from dysregulated TGF-β activity. TGF-β cytokines act through various small GTPases such as RhoA and Rac1, which are implicated in valve disease and development (Figure 1). RhoA is a mechano-sensitive GTPase that acts complementary to Rac to control cell migration, differentiation, and proliferation. Filamin-A (FlnA) point mutations in mice, responsible for X-linked myxomatous valve disease (57), deregulate the balance between RhoA and Rac1 in favor of RhoA, altering downstream trafficking of β1 integrins (58) resulting in a myoxomatous phenotype by 2 months of age. For more information on GTPases, see section on Integrated Mechanotransduction at the end. FlnA mutations increase Erk signaling, a non canonical TGF-β driven kinase, which is present in mouse models of MFS (59). In murine aortic valves with an elastogenic defect, mice had latent hemodynamic AV disease from increased Erk1/2 activation, ECM disorganization, and inflammation (60). Both these mutant mice and aged mice display stiffened ECM, fibrosis, cell adhesion and fibronectin alterations, increased collagen expression, and decreased LTBP signaling (60) suggesting a similar mechanism may be driving integrin signaling in MVD.

## Cilia

Primary cilia are solitary microtubule structures consisting of a basal body and projecting axoneme “antenna.” The axoneme senses the external environment and coordinates various signaling pathways, such as TGF-β (61) and calcium sinks (62), indicating a mechanosensory role (63). Primary cilia defects have been linked to various congenital cardiovascular diseases, such as heterotaxy and atrioventricular septal defects (64–66). Cilia are strongly expressed between stages E11.5 and E17.5 on the outflow tract cushions in aortic valvulogenesis, while they are lost in adult VICs (67).

Primary cilia restrain ECM expression during development and remodeling such that ablation of primary cilia during aortic valvulogenesis results in highly penetrant bicuspid valve phenotype (67). Primary cilia loss in arterial ECs sensitizes them toward BMP mediated osteogenic differentiation (68), inflammatory gene expression, and decreased eNOS activity (69). Exome sequencing of chemically mutagenized mice revealed mutations in 61 recessive congenital heart disease genes, 34 of them cilia related (66); cilia axoneme mutants caused outflow tract and atrioventricular septation (70). In polycystic kidney disease (PKD), a genetic disorder with TGF-β mediated abnormalities, there is a 10-fold increase of mitral valve prolapse tied to defective protein localization in the primary cilia (71–73). Mitral insufficiency has been seen in infantile nephronophthisis (74), structural defects in Ellis–van Creveld syndrome (75, 76), severe mitral regurgitation and structural defects in Kartagener’s syndrome (77, 78), and rheumatic valvular insufficiency in Bardet–Biedl syndrome (79).

## Ion Channels

**Figure 2:**
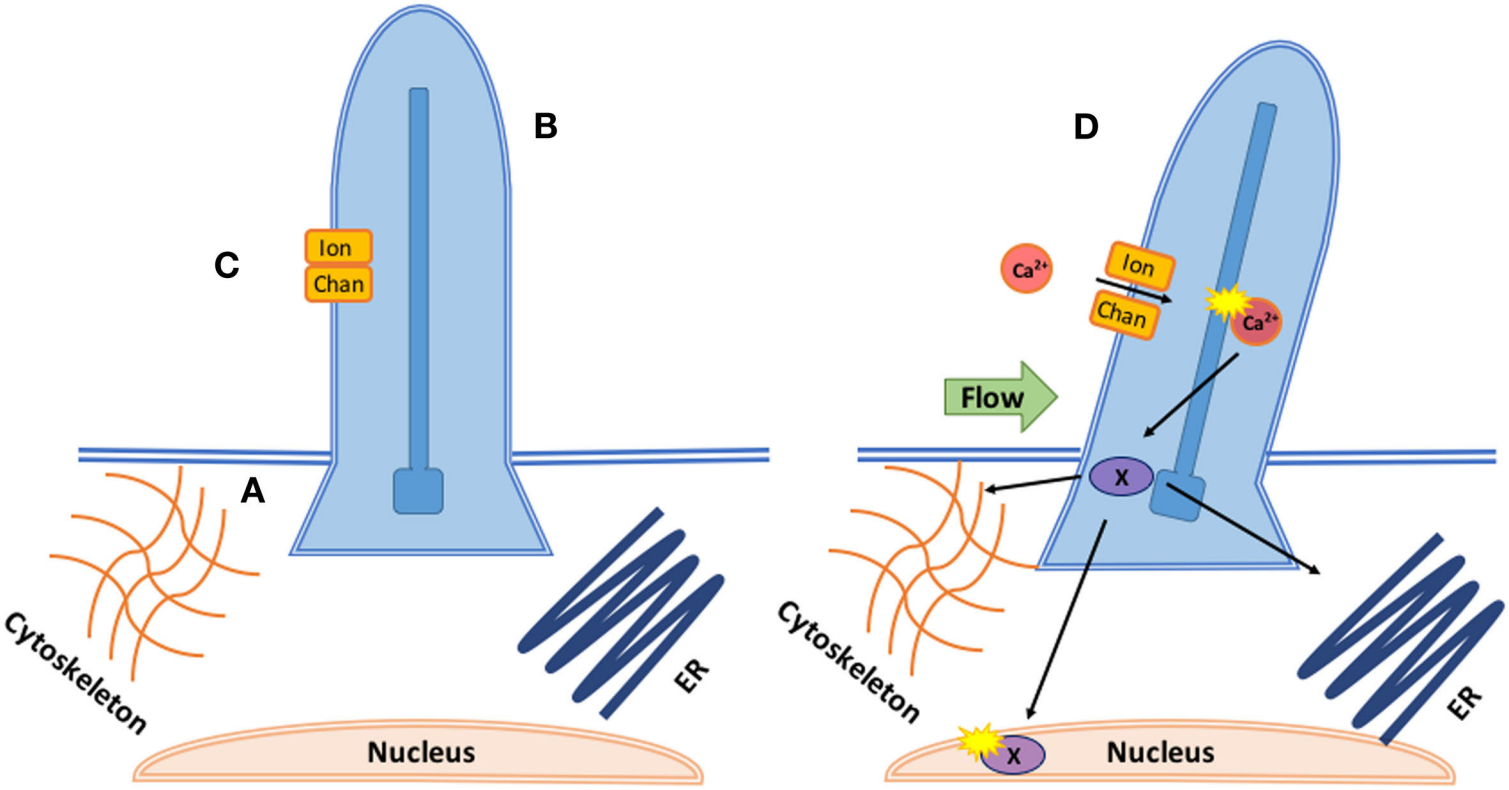
Coupled mechanotransduction of cilia and ion channels. A. The basal body is a modified centriole that sits at the bottom of the cilia and provides the origin point for new cilia. They provide a symmetric template for the axoneme structure and dictate the position and orientation of the cilia, ensuring correct cilia-driven fluid flows and response to flow. B. The axoneme is the most prominent component of cilia consisting of nine microtubules. Its major function is cell signaling as the axoneme senses and coordinates mechanical and chemical responses, bending the cilia and altering downstream signaling. Intraflagellar transport brings cargo into the cilia along the axoneme with kinesin and out with dynein. C. In response to axoneme bending, stretch or calcium responsive ion channels, such as the polycystins (polycystic kidney diseases), open, allowing for calcium- or ATP-dependent signaling to occur inside the ciliary body. D. In response to calcium influx through the ion channel, downstream transcription factors (X) are phosphorylated and translocated to interact with the cytoskeleton, nucleus, and endoplasmic reticulum.

Mechano-sensitive channels (MCs) are a class of membrane ion channels that detect and respond to force, converting it into electrical or biochemical signals (80, 81). There is increasing evidence MCs play a key role in regulating endothelial response to shear flow (82–84). Cilia coupled with calcium channels (Figure 2) transduce shear stress during zebrafish valvulogenesis; endothelial cilia deflect with blood flow correlating to expression of calcium channel gene polycystin-2 (PKD2), increasing endothelial calcium levels, and altering vascular formation (85). Cilia response is mediated by transient receptor channels such as Trpv4 and Trpp2 which are expressed during valve development (86). mRNA expression of Piezo1, a mechanically activated cation channel, has been seen in murine hearts (87) while its loss in ECs causes stress fiber and cell orientation (88) deficits in response to shear stress, profound vascular defects, and embryonic lethality within days of the heart beating (89).

Malfunction of MCs results in broad cardiovascular pathology such as arrthymias (90), hypertension (91), and PKD (92). PKD2 is localized to the cilia in vascular ECs. In mouse embryos it is required for right-left axis determination with knockouts displaying severe cardiac structural defects by E18 (93). PKD2 is mutated in PKD and murine mutants lose the ability to generate nitric oxide (NO) in response to shear flow which may promote high blood pressure (94). PKD2 defects may prolong channel activity by preventing calcium from leaving small compartments, such as cilia (95). Prolongation of the QT interval has been associated with myxomatous mitral valve related sudden cardiac death (96, 97). Mutations in sodium voltage-gated channel V account for 5–10% of long QT cases and have been comorbid with desmoplakin mutations, a protein responsible for mechanical coupling of cardiac myocytes with known overlap in channelopathies (97, 98). Oscillatory flow stimulates klf2a expression, a key transcription factor in valvulogenesis, and knockdown results in dysfunctional or absent leaflet formation (15). Oscillatory flow through Trpv4 and Trpp2 (99) modulate the endocardial calcium response and control klf2a expression in zebrafish, with absence of either resulting in severe valve defects. klf2a misexpression during angiogenesis occurs in the absence of flow, with downregulation of β1 integrin rescuing overgrowth and maintaining endothelial quiescence (100).

## Caveolae

Caveolae are small plasma membrane invaginations made up of Caveolin (Cav) and Cavin proteins, glycosphingolipids, and cholesterol. Caveolae respond to mechanical stress by flattening into the membrane, increasing surface area to relieve tension, while confining receptors and signaling molecules (101, 102). Caveolae participate in a dynamic cycle of flattening and reassembly in response to mechanical stress independent the actin cytoskeleton. In vascular smooth muscle cells (103), cardiomyocytes (104), and aortic ECs, translocation of Cav1 to non-caveolar membrane domains during flattening is required for strain and flow induced Erk expression (105). Rho and Rac GTPases (104), Src (106) and MAP kinases (107), and calcium (108) expression are also modulated by caveolae mechanotransduction.

Genomic analysis in canine myxomatous valve disease identified caveolar mediated endocytosis as a canonical pathway relevant to MVD (12). This pathway controls EC growth and migration through endocytosis of cholesterol-enriched membrane microdomain (CEMM) internalization when integrins are uncoupled during cell detachment from the ECM. Integrins target Rac to CEMMs where it interacts with downstream effectors to induce signaling (109, 110). In caveolin-1 knockdowns, TGF-β, fibroblast activation, and collagen gene expression increases in human lung fibroblasts (111). In canines with chordal rupture induced mitral regurgitation, caveolar invagination decreased Erk signaling, regulating hypertrophic remodeling in response to volume overload (112). Positive caveolin staining and caveolae structures have been seen on aortic VECs (113) and may be conserved in mitral valves.

## Glycocalyx

The glycocalyx (GC) are abundant proteoglycan complexes that cover the surface of ECs and maintain endothelial barrier integrity. They are composed of the syndecan, a transmembrane core protein, and membrane anchored GAGs (114). GC control NO production (115) in vascular ECs by transducing shear stress to the cytoskeleton (116, 117) resulting in intracellular signaling and NO production (118, 119). Breakdown of the GC results in dissolution of tight junctions (120) and production of NO is dependent on calcium intake from TRP channels (121).

Syndecans (Sdcs) are members of a proteoglycan family of adhesion transmembrane receptors (122, 123). There are four mammalian Sdcs that bind to ECM, cell adhesion molecules, and growth factors (43). While no Sdcs are expressed in healthy aortic or mitral VICs (43), Sdc1 is strongly expressed on the vascular EC surface (124) and GCs are broadly expressed on the mitral endothelium in hypercholesterolemic rabbits (125). GCs and Sdcs are implicated in inflammatory (126, 127) and vascular diseases in the context of heart failure (128–130), myocardial dysfunction (131), and myocardial infarct (132). Sdc1-null mice with myocardial infarction display enhanced endothelial adhesion, trans endothelial migration of inflammatory cells, matrix remodeling, and fibrosis (133) as well as attenuated angiotensin II-induced dysfunction (134). Oxidized LDL cholesterol degrades GCs and enhances adherence of leukocytes to the endothelial surface in mouse vascular models (135). Immune involvement provides a potential avenue to MVD given the autoimmune role in rheumatic valve disease.

## Nuclear

Many mechanosensing modalities are physically coupled to the cytoskeleton filaments which in turn link to nuclear scaffolds, chromatin, and nuclear DNA (136–138). Forces applied to the cell surface cause structural changes to the nucleus (139, 140). As such, the nuclear aspect ratio (NAR) can be used as an index of cellular deformation due to the correlating deformation and directionality of the nucleus to the cell. In the mitral valve, NAR analysis determined VICs in the fibrosa and ventricularis layers deform more than the atrialis and spongiosa (141). MVICs also display cytoplasmic uncoupling from nuclear deformation under hyper-physiological strain levels (142) which may have phenotype and ECM remodeling consequences.

Lamins, nuclear intermediate filaments, are dense protein networks capable of forming stable structures within the nucleoplasm and have a crucial role in DNA/RNA synthesis and transcription (137). Dilated cardiomyopathy (143, 144) a laminopathy, causes volume overload and functional mitral regurgitation. Lamin A/C mutant mouse cells have impaired activation of mechano-sensitive transcription factor MRTF-A which causes cardiac myofibroblastic differentiation (145, 146) and activates vinculin and actin (147). Linker Nucleuskeleton and Cytoskeleton (LINC) proteins are key mechanotransductive structures between the cytoskeleton and nucleus. They include nesprin which connects LINC to the cytoskeleton and SUN which anchors LINC in the nucleus through lamin interactions and chromatin binding proteins (148). Nesprin is subject to actin-myosin mediated tension in adherent fibroblasts, which is reduced in fibroblasts from Hutchinson–Gilford progeria patients, a multisystem laminopathy (149). Nesprin also interacts with common intracellular signaling pathways such as Erk1/2 (67) and β catenin (150). Nesprin knockdown in ECs cripples nuclear deformation and cell orientation during cyclic strain, but increases focal adhesions (151). Nesprin knockout cells have altered morphology, polarization, and migration (152).

## 5-HT Serotonin

Multi-valve pathology (153) is seen after exposure to serotonergic drugs fenfluramine, dexfenfluramine, ergotamine, and methysergide (154) as well as ergot-derived dopamine agonists pergolide (155), cabergoline (156), and bromocriptine (157). Fenfluramine binds to serotonin or 5-hydroxytryptamine (5-HT) receptors 5-HT_2A_, 5-HT_2B_, and 5-HT_2C_ with porcine aortic and mitral VICs expressing 5-HT_2A_ and 5-HT_2B_ receptor transcripts, suggesting valve fibrosis (158) after exposure to fenfluramine, ergot drugs, and 5-HT is a result of 5-HT_2A_ and 5-HT_2B_ stimulation. In ligand screening studies 5-HT_2B_ is the commonly activated serotonin receptor of drugs associated with valvular heart disease (159) with myxomatous canine valves upregulating 5-HT_2B_ receptor mRNA (160) and proteins (161). The 5-HT_2B_ receptor is required for heart development (162) regulating differentiation and proliferation of cardiac tissue; 5-HT transporter deficient mice develop cardiac fibrosis, and valvulopathy (163).

5-HT_2B_ increases MVIC proliferation and ECM production through common mechano-active signaling modalities. 5-HT_2B_ receptor activation increases MAPK activity through Erk1/2 (164, 165) as well as Src family kinases (166), resulting in cell proliferation, while addition of 5-HT to canine MVIC cultures increases collagen and GAG synthesis through H-proline and H-glucosamine incorporation respectively (164). Cross-talk may occur between the TGF-β and 5-HT pathways under elevated mechanical stresses. During atrioventricular valve development in chick embryos, 5-HT induces pathological modeling effects through a TGF-β3-dependent mechanism causing tissue stiffening, contractile gene expression, and collagen expression (167). In myxomatous mitral valves, 5-HT_2B_ receptor expression is co-localized with αSMA expression (168); neonatal rat cardiac fibroblasts treated with 5-HT upregulated αSMA expression marking fibroblast differentiation and TGF-β signaling (169). AVICs treated with 5-HT show increased TGF-β1 and 5-HT_2A_ (170) expression while serotonin transporter (SERT) knockout embryonic mice increased expression of TGF-β1, αSMA, and 5-HT_2A_ in the whole heart (171). At the tissue scale, treating an AVIC seeded construct with a 5-HT_2B_ agonist acutely decreases tone generation of the cells, tissue alignment, and increases the tensile modulus along the primary fiber alignment axis (172). Similar mechanisms may be at play in 5-HT-related MVD.

**Figure 3:**
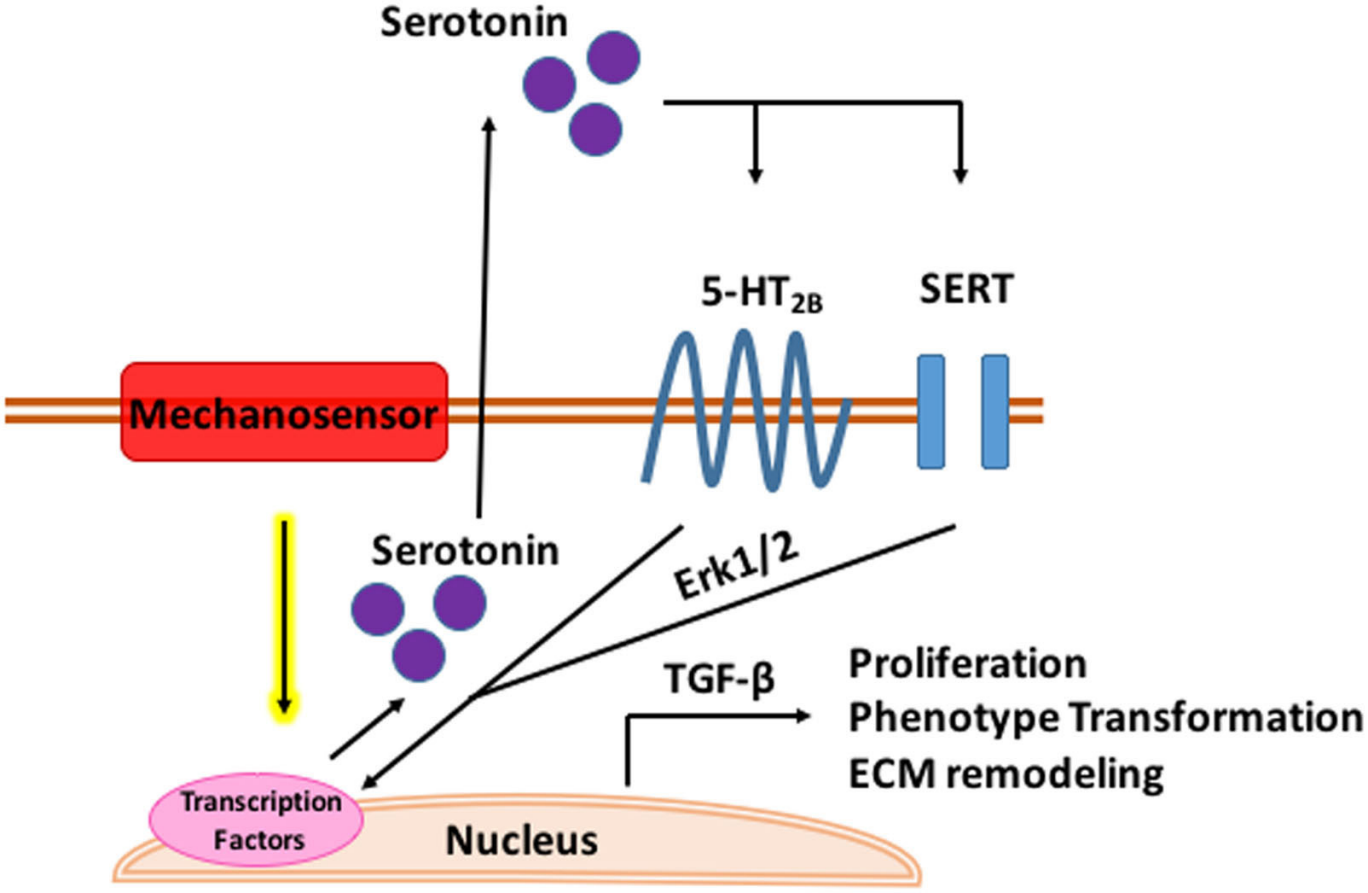
Mechanomodulation of mitral valve disease through serotonin. Tensile strain upregulates serotonin synthesis through a mechanosensory mechanism. Serotonin interacts with the serotonin type 2B receptor and serotonin transporter (SERT) in the mitral valve activating Erk1/2 through G-protein stimulation. Erk1/2 is phosphorylated in the nucleus where it induces TGF-β signaling and transcription of genes mediating myxomatous disease.

While 5-HT alters the MV microenvironment and global valve mechanics, it may also be a direct mechanomodulator as proposed in Figure 3 below. In both aortic banded rats and neonatal rat cardiomyocytes, mechanical stress enhances 5-HT_2B_ signaling in ventricular models of pressure induced cardiomyopathy (173). Serotonin induced a positive inotropic response in the papillary muscles and increased 5-HT_2B_ receptor expression in hypertrophic rats with post infarction heart failure which correlated to degree of hypertrophy (174). Cyclic stretch upregulates 5-HT_2A_ and 5-HT_2B_ receptor expression in porcine aortic valve cusps causing AVIC proliferation and ECM remodeling (175). Cell proliferation, collagen synthesis, and tissue stiffness in response to cyclic stretch seem to be specifically modulated by the 5-HT_2A_ receptor in the aortic valve (176) while unstrained *in vitro* experiments in MVs implicate the 5-HT_2B_ receptor. Static and cyclic strain increase expression of myxomatous effector proteins, chondrogenic markers, and markers of the myofibroblastic phenotype compared to unstrained controls in myxomatous canine MVs (177). Interestingly, in both strain conditions, expression of serotoninsynthetic enzymes increased with higher serotonin levels in the media of cyclically strained valves suggesting mitral valves are capable of local serotonin synthesis and may be mechanically modulated (177). Myofibroblastic phenotype markers, matrix catabolic enzymes, cathepsins, matrix metalloproteases, and GAGS increased with increasing cyclic strain in cultured sheep MVs with serotonin present in the media of cyclically strained valves with concentration correlating to percent strain; inhibition of serotonin reduced these strain mediated protein expression patterns (178).

## Integrated Mechanotransduction

**Figure 4:**
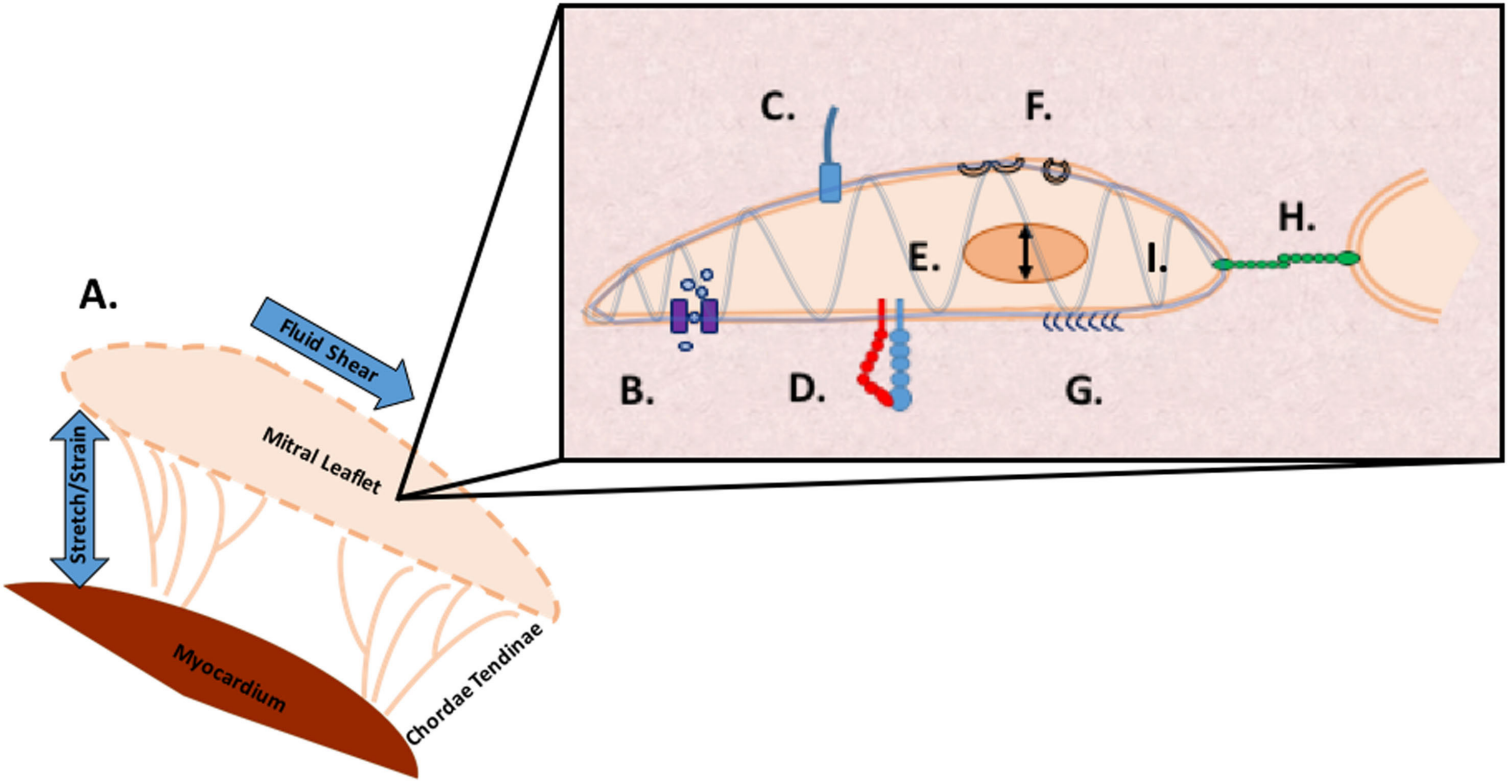
Methods of mechanosensing in the mitral valve. A. At a global level, the valve is subjected to flexure as the valve opens, shear as the blood flows through the valve, flexure as the valve closes, and tension as the valve seals shut to prevent regurgitation. At a microscopic level, mechanotransduction converts these extracellular forces into intracellular signaling through multiple cellular apparatuses. B. Mechano-sensitive ion channels convert mechanical force exerted on the cell membrane into electrical or biochemical signals. C. The axoneme of primary cilia convert extracellular cues into various signaling pathways as well as coupling transduction with voltage-gated channels D. Integrins are the main receptors connecting the cytoskeleton to the extracellular matrix (ECM) and transmit mechanical stress across the plasma membrane E. In nuclear deformation physical force is transmitted across the nuclear envelope to the nuclear interior where they modulate gene expression from physical deformation of genetic material F. Caveolae flatten into the plasma membrane when stimulated by cell-surface tension, relieving tension and physically sequestering proteins, growth hormones, and cytokines G. The glycocalyx transmits fluid shear stress to the cell through core proteins which connect to the actin cytoskeleton and cell membrane mediating cell signaling H. Cadherins are cell adhesion proteins that create zipper like structures at cell junctions to maintain stable intercellular adhesion and mechanical coupling between cells and the adherens junction to transform mechanical to chemical signals as well as interacting with integrins through actin filaments I. Directly or indirectly, the load bearing cytoskeleton is common to the various mechanosensing modalities. Often clustering at focal adhesions, the cytoskeleton rapidly transmits ECM stimulus into cellular response through actin filament reorganization.

It is likely individual methods of mechanotransduction work in concert through common signaling pathways. Multi faceted proteins such as small GTPases coupled with an integrated framework, such as the cytoskeleton, implicate a coordinated sensing and transduction network of shared, simultaneous components as illustrated in Figure 4.

Both RhoA and Rac GTPases mediate endothelial–mesenchymal transition during valvulogenesis (179, 180), while in adult VICs RhoA regulates actin cytoskeleton and stress fiber formation as Rac regulates cell–cell adhesion, actin polymerization, lamellae protrustion, and cytoskeletal polarity (181). Altering the actin network geometry by overexpressing Rac1 GTPase so precursor actin bundles are suppressed at free borders, changes adherens junction shape, and increases lamellae protrusions (182, 183). RhoA signaling couples cadherin based adhesion with actimyosin contractility (184). Rac1 and RhoA interact in a spatiotemporal manner with adherens junction proteins to coordinate opening and closing of endothelial junctions (185). FilGAP, a Rac GTPase-activating Protein, binds FlnA to control actin remodeling (186) and is present at focal adhesions but more directly present at cytoskeletal interfaces where FlnA and the β integrin cytoplasmic tail interact to form a binding pocket for opposing β strands (187, 188). FlnA is an actin binding protein widely expressed during valvulogenesis, which anchors transmembrane proteins to the cytoskeleton and mediates remodeling events in response to stimulus.

In both embryonic and adult VICs, a quiescent phenotype is maintained when they are cultured in unstressed collagen hydrogels; however, contractile expression, TGF-β, and matrix remodeling are upregulated in response to mechanical tension (55, 189). During development, this quiescent phenotype transition is governed by decreasing αSMA following decreased RhoA-GTPase expression (11, 190). Cyclic stretch of embryonic valve progenitor cells activates RhoA in acute response to the mechanical stimulus and is later switched to chronic Rac1 activation through FilGAP (191). RhoA mediates myofibroblastic activation during this acute signaling while chronic cyclic strain deactivates RhoA, enabling Rac1 to compact the matrix. Mutations in FlnA are responsible for X-linked myxomatous valve disease (192) by weakening FilGAP binding (193) and disrupting GTPase regulation (58) which alters cytoskeletal remodeling ability. Rac-1 knockdown in embryonic kidney cells abrogated PKD1-mediated signaling suggesting a critical role for small GTPases in PKD, providing insight into ciliary and voltage-gated signaling (194). In Bardet–Biedl syndrome, RhoA levels are upregulated but treatment of mutant cells with RhoA inhibitors restores cilia length and number as well as actin cytoskeleton integrity (195). In vascular SMC, 5-HT induced mitogenesis relies on Rho-mediated translocation of Erk1/2 (196) and induces Smad activation in bovine and human pulmonary artery SMCs *via* RhoA (197). 5-HT potentiates TGF-β3 expression in cushions which then induces contractile gene expression through RhoA (167).

The cytoskeleton provides an integrated framework for communication by physically connecting distant parts of the cell (145), rapidly transmitting mechanical information and modulating signal transduction through posttranslational modification, remodeling, and reorganization. Mechanical activation of Src 50 µm from the point of force application in vascular smooth muscle cells takes less than 300 ms through actin stress fibers, orders of magnitude faster than reaction-diffusion signaling cascades (145, 198). Disrupting actin filaments (199) as well as relieving stress fiber prestress (200) impairs rapid long distance mechanotransduction. Association with cadherins and integrins produces a critical interface through which actin filaments are exposed to forces from the ECM. Integrins and cadherins share similar mechanotransductive mechanisms in their interactions with the actin cytoskeleton, recruitment of common adhesion components, and extensive cross-talk (200, 201). Both integrins and cadherins stimulate Rho and Rac GTPases resulting in cytoskeleton remodeling in response to adhesion (201, 202).

The actin cytoskeleton provides structural stability to GC in ECs under shear stress (203). Depolymerizing actin weakens the anchoring strength of core proteins that support the GC such that the GC layer is ablated under shear stress; this is potentially due to altered mechanotransduction (203). Caveolae associate and align with stress fibers (204, 205) through FlnA actin binding domains. Knock down of FlnA increases the lateral movement of Cav1 and reduces stress fiber alignment of the caveolae (206). Inhibiting actin polymerization increases the abundance of caveolar rosettes and increases Cav1 (207, 208) clustering while increasing stress fiber formation decreases caveolar rosettes (209). Caveolae, specifically Cav1 interactions (210, 211), regulate RhoA-mediated actomyosin contractility (209). Cav1 and RhoA are localized to the same membrane invaginations (212), physically interacting to induce cytoskeletal reorganization in response to force (104). Like FlnA mutations, alterations to the ECM change cytoskeletal structure and function which can result in pathological signaling and remodeling. Erk activity specifically localizes to regions of matrix metallopeptidase 2 expression (213), an ECM degrading enzyme, which is significantly increased in clinical patients with floppy mitral valves and mitral valve prolapse (214). A variety of collagen mutations result in mitral valve prolapse, aortic root dilation, and a host of structural defects (215, 216).

The mitral valve exists in a complex environment where global mechanical deformation alters cell phenotype and ECM remodeling (217) in the microenvironment in a synergistic and reciprocating fashion. It is increasingly apparent that multiple mechanobiological regulatory modalities exist and are interconnected through shared components. Much like our five senses, multiple methods of mechanosensing coexist in the same cell, interacting with each other and the environment. In cells with a disrupted sense, mechanical stimulus may seem preferentially potent in one sense compared to a wild-type cell, causing pathological signaling and remodeling. The interconnected pathways and frameworks of mechanotransduction can be thought of as a network in search of homeostasis; superior treatments may seek to rebalance the network instead of focusing on a solitary gene or protein defect. Increasing our understanding of how cells interact with their environment through mechanosensing and mechanotransduction provides potential therapeutic targets in valve disease by altering the environment, cellular perception of the environment, or communication with the environment in a profound and regenerative manner.

## Author Contributions

JB suggested the subject of the review, recommended resources, direction of the review, suggested types of figures to include, and provided extensive editing. LP is a Ph.D. candidate in JB’s group and based on the recommendations of JB did an extensive literature review, drafted the article, created figures, and charted the direction and subject matter contained in the review.

## Conflict of Interest Statement

The authors declare that the research was conducted in the absence of any commercial or financial relationships that could be construed as a potential conflict of interest.
